# Usefulness of Different Imaging Modalities in Evaluation of Patients with Non-Alcoholic Fatty Liver Disease

**DOI:** 10.3390/biomedicines8090298

**Published:** 2020-08-21

**Authors:** Karolina Grąt, Michał Grąt, Olgierd Rowiński

**Affiliations:** 1 Second Department of Clinical Radiology, Medical University of Warsaw, 02-097 Warsaw, Poland; olgierd.rowinski@wum.edu.pl; 2 Department of General, Transplant and Liver Surgery, Medical University of Warsaw, 02-097 Warsaw, Poland; michal.grat@gmail.com

**Keywords:** NASH, NAFLD, liver stiffness, liver steatosis, controlled attenuation parameter, transient elastography, MRI PDFF, MR spectroscopy

## Abstract

Non-alcoholic fatty liver disease (NAFLD) and non-alcoholic steatohepatitis (NASH) are becoming some of the major health problems in well-developed countries, together with the increasing prevalence of obesity, metabolic syndrome, and all of their systemic complications. As the future prognoses are even more disturbing and point toward further increase in population affected with NAFLD/NASH, there is an urgent need for widely available and reliable diagnostic methods. Consensus on a non-invasive, accurate diagnostic modality for the use in ongoing clinical trials is also required, particularly considering a current lack of any registered drug for the treatment of NAFLD/NASH. The aim of this narrative review was to present current information on methods used to assess liver steatosis and fibrosis. There are several imaging modalities for the assessment of hepatic steatosis ranging from simple density analysis by computed tomography or conventional B-mode ultrasound to magnetic resonance spectroscopy (MRS), magnetic resonance imaging proton density fat fraction (MRI-PDFF) or controlled attenuation parameter (CAP). Fibrosis stage can be assessed by magnetic resonance elastography (MRE) or different ultrasound-based techniques: transient elastography (TE), shear-wave elastography (SWE) and acoustic radiation force impulse (ARFI). Although all of these methods have been validated against liver biopsy as the reference standard and provided good accuracy, the MRS and MRI-PDFF currently outperform other methods in terms of diagnosis of steatosis, and MRE in terms of evaluation of fibrosis.

## 1. Introduction

**Figure 1:**
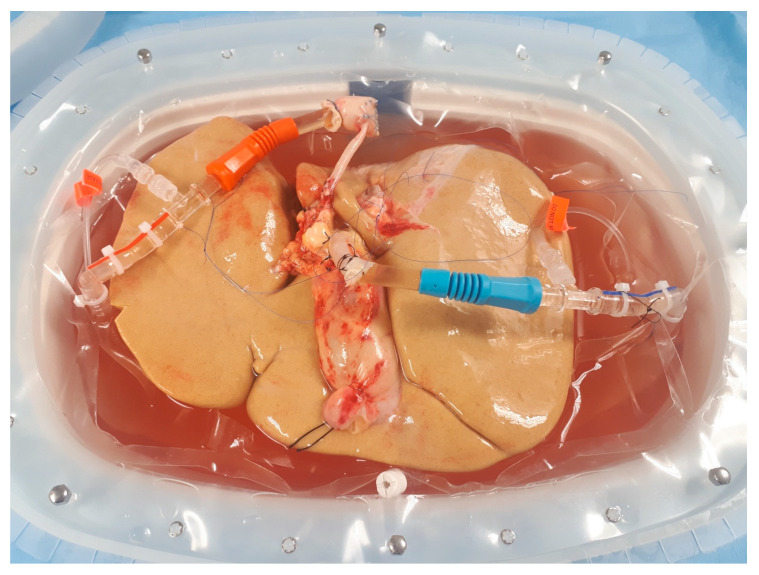
Liver allograft with extensive steatosis undergoing hypothermic oxygenated machine perfusion. Image from the authors’ department.

Non-alcoholic fatty liver disease (NAFLD) is a chronic liver condition with globally increasing incidence rates. It is associated with the worldwide increasing prevalence of overweight, obesity, and metabolic syndrome, and is becoming one of the most important health system issues in the developed countries [1,2]. NAFLD, which is defined as an excessive accumulation of fat in hepatocytes, may progress to non-alcoholic steatohepatitis (NASH), and further into fibrosis, with all of its complications—the development of liver cirrhosis, portal hypertension, and hepatocellular carcinoma [3]. It has also been proven that patients with NAFLD have a higher risk for the occurrence of cardiovascular incidents [4,5,6] and increased risk for developing chronic kidney disease [7]. Remarkably, it is estimated that up to 35% of citizens of the United States of America (USA) are affected by NAFLD, which makes it the most prevalent liver disease in the USA [8]. Accordingly, end-stage liver disease in the course of NASH has already become one of the most common indications for liver transplantation in the USA. Importantly, recent analyses predict a further increase in the prevalence of NASH, with more than 60% of Americans estimated to be affected by the year 2030 [9,10]. Therefore, major effects on the access to liver transplantation, pre-transplant-mortality, wait-list dynamics, and the general outcomes of liver transplant recipients are expected. As grafts with excessive fat accumulation are not suitable for transplantation, increasing prevalence of NASH influences the donor pool. Recently various techniques, such as hypothermic oxygenated machine perfusion, have been proposed to improve the quality of grafts (Figure 1).

Histopathological evaluation of liver biopsy still remains the gold standard for the diagnosis of NAFLD and NASH. Liver biopsy is also used as the reference standard for the assessment of other methods, despite several disadvantages. Due to the invasive character, it is associated with the risk of potentially severe complications. Further, the costs of frequent liver biopsies are relatively high [11,12,13]. What is more, some authors suggest that the results might be misleading, as the specimen may not be representative of the whole organ, especially in relatively benign changes. Therefore, alternative non-invasive methods of more representative assessment of liver steatosis have been proposed and validated in the recent years. Ideally, those should be non-invasive, widely available and cost-effective. As there are many ongoing clinical trials on new therapies for patients with NAFLD and NASH, new methods should be particularly characterized by the clinical utility to perform regular follow-up in order to verify treatment results. Moreover, it should also enable precise quantitative evaluation of the current status of liver parenchyma.

This is a narrative review aimed at presenting the current information on various methods used for the assessment of liver steatosis and fibrosis. Further, available data on their prognostic role in patients with NAFLD are discussed. The choice of the articles for this narrative review was made after evaluation by the authors following screening of abstracts in the PUBMED database, using the following search terms: “NASH diagnosis”, “NAFLD diagnosis”, “liver steatosis”, “liver spectroscopy”, “PDFF”, “proton density fat fraction”, “computed tomography liver steatosis”, “liver elastography”, “controlled attenuation parameter”.

## 2. Techniques Using Computed Tomography

**Figure 2:**
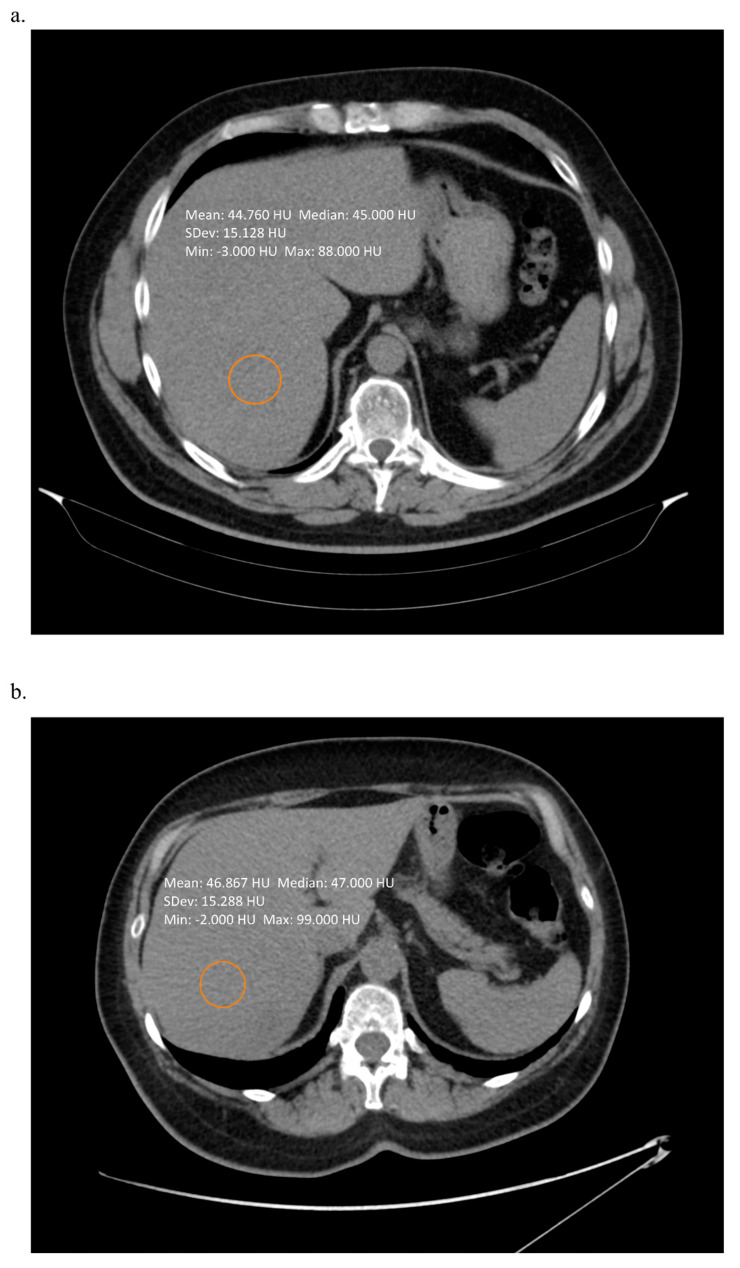
Non-contrast enhanced computed tomography scan of a patient with (**a**) 60% of hepatic steatosis (**b**) 80% of hepatic steatosis. In both cases simple measurement of the liver density was not suggestive on such severe changes. Images from the authors’ department.

Computed tomography (CT) scans provide valuable information on the extent of liver steatosis, basing on the analyses of the organ density. The most basic techniques, which are simply based either on the measurement of the liver density on non-contrast enhanced scans or comparisons between the density of the liver to the density of the spleen [14], can accurately detect liver steatosis exceeding 20%, but fail to provide sufficient accuracy in patients with hepatic steatosis of lesser extent [15]. In a metanalysis performed by Bohte et al., the overall specificity of CT in the diagnosis of any liver steatosis (with biopsy used as the reference standard) was as low as 46–72%, with the lowest accuracy observed for mild forms of fat accumulation. Currently, there are no algorithms for precise and accurate quantitative assessment of fat content in the liver tissue. Therefore, the use of CT seems rather limited to general stratification of patients and it is not a suitable diagnostic modality in long term follow-up; for example, in patients undergoing treatment for NAFLD/NASH. Even extensive steatosis of more than 50% of hepatocytes can present with a density higher than the standard cut-off of 40 Hounsfield units, as shown on Figure 2.

Nevertheless, several variations of the standard scanning technique have been proposed in order to enhance the diagnostic accuracy of the CT scans. One is the use of a standardized calibration phantom, placed beneath the patients back during the CT examination. In a recent study from 2020, Guo et al. provided evidence that the utilization of this protocol enables the calculation of hepatic steatosis far more accurately, with sensitivity and specificity of 76% and 85%, respectively, for the detection of mild steatosis (involving at least 5% of hepatocytes), and 85% and 98%, respectively, for the detection of moderate steatosis (involving at least 14% of hepatocytes) [16]. The clinical usefulness of this method is especially supported by relatively high positive and negative predictive values of 78% and 83%, respectively, for mild steatosis, and 82% and 97%, respectively, for moderate steatosis. Importantly, this technique needs to be adjusted for particular type of CT scanner, as the basic liver density in Hounsfield units may differ between different manufacturers.

Computed tomography fails to detect early stages of liver fibrosis, and can only show signs of advanced stages, for example nodular shape of the liver, evidence of portal hypertension etc. Nevertheless, some authors have proposed advanced algorithms for the assessment of liver fibrosis on CT scans—for example, analysis of liver texture [17], analysis of the nodularity of the liver surface [18,19] or incorporating data from the CT into an multiparametric tool (data from the CT scans combined with laboratory tests) [20]. All of these have succeeded in providing good accuracy, especially in higher stages of fibrosis. In a study of 556 patients, Lubner et al. created a model based on a combination of four factors indicating liver texture, which provided and area under the receiver operating curve of 0.82, 0.82 and 0.86 for the diagnosis of any (≥F1), significant (≥F2), and severe (≥F3) fibrosis, respectively [17]. While liver surface nodularity analysis provided excellent areas under the curve for the detection of both mild and severe fibrosis, the findings seem limited by almost identical cut-off values of 2.8, 2.77 and 2.9 for significant (≥F2) fibrosis, severe (≥F3) fibrosis and cirrhosis (F4), respectively [19]. Although the corresponding cut-offs were more separated in another study on liver surface nodularity, the overlapping values in patients with different stages of fibrosis remained as a major limitation of its clinical utility [18]. Regarding inclusion of computed tomography features into a multiparametric model, a combination of nine factors assessed on CT with Fibrosis-4 score [21] and aspartate transaminase-to-platelets ratio index [22] was proposed. It resulted in moderate improvement in the diagnostic accuracy with respect to mild (F1), moderate (F2), and severe (F3) fibrosis in a study performed on 469 patients, yet with hepatitis C virus infection [20].

In addition to evaluation of the status of liver parenchyma, many studies have shown that computed tomography is a good diagnostic modality for the purpose of analyzing patients’ body composition, in particular the amount of visceral and subcutaneous fat, and this may be especially important in patients dealing with obesity (which is the case in the vast majority of NASH/NAFLD patients), as the simple Body Mass Index (BMI) has been shown to provide insufficient accuracy [23]. For instance, in a study performed on 76 patients with liver cirrhosis, more than 20% of patients with normal body mass index had an increased amount of adipose tissue, whereas 40% of overweight patients were found to have normal amount of adipose tissue [24]. The amount of fat tissue may be measured on a single CT scan (usually on the level of the body of the third lumbar vertebra), based on the threshold of Hounsfield units, which is a fast and easy technique. There is no consensus on the optimal cut-off points. What is more, some authors define their proposed cut-off values as simple area (not adjusted for the height), which can significantly impair wide use in different populations. Notably, even in homogeneous populations, the cut-off values differ significantly; for example, in different studies on the Korean population, authors proposed values for the visceral fat area ranging from 92.6 cm^2^ to 140 cm^2^ for men and from 75 to 100 cm^2^ for women [25,26,27,28,29]. The measurement can be also propagated on the whole abdominal scans (manually or by using advanced algorithms) [30,31] to calculate the whole visceral fat tissue volume. It is, however, unclear, whether evaluation of the latter provides any benefits over single-scan assessments and this should be elucidated further, yet the arguments for the use of volume over surface analyses include: independence from bowel movement and patients’ breathing, individual constitutional characteristics or bowel capacity. Previous studies have shown that excess amounts of fat tissue (both visceral and subcutaneous) play an important role in carcinogenesis, and also in hepatocellular carcinoma (HCC) [24,32]. Therefore, it seems reasonable to routinely evaluate the amount of fat tissue in patients undergoing CT scans, especially in a group of NASH patients, who can progress to liver cirrhosis and develop HCC.

Obviously, one of the biggest disadvantages of CT scans is patient exposure to radiation, which precludes its regular, repeated, and life-long use, for example during regular follow-up for the assessment of NAFLD progression. However, CT scans are much more available than the MR scans and their cost is lower. What is more important, many patients are undergoing computed tomography for other indications, and the assessment of hepatic steatosis can be performed at the same time to provide additional, valuable clinical information.

## 3. Magnetic Resonance Imaging Techniques

New techniques in magnetic resonance imaging (MRI) have been proven to provide good specificity and sensitivity in detecting liver steatosis and are now the reference standard to which other diagnostic imaging modalities should be compared. The most promising method with excellent results and—very importantly—standard examination technique, not requiring any additional equipment, is the chemical shift-encoded MRI proton density fat fraction (MRI-PDFF). The examination can be performed on both 1.5 T and 3.0 T scanners [33,34], which are widely available at most hospitals. The technique is usually based on acquisition of 6-echo chemical-shift-encoded gradient-echo sequences, but it has been shown that acquisition of less—for example 2 or 4 echo sequences—provides nearly identical results. However, in some cases—for instance in patients with iron deposition in the liver parenchyma—the results may be influenced in case of dual-echo or triple-echo methods, which is not the case with multi-echo [35]. MRI-PDFF method has been validated in comparison to magnetic resonance spectroscopy [34,36,37,38] and also provides excellent intra-examination repeatability [34] and inter-examination repeatability [39]. Importantly, the results are highly comparable among different fields and scanner manufacturers [40].

**Figure 3:**
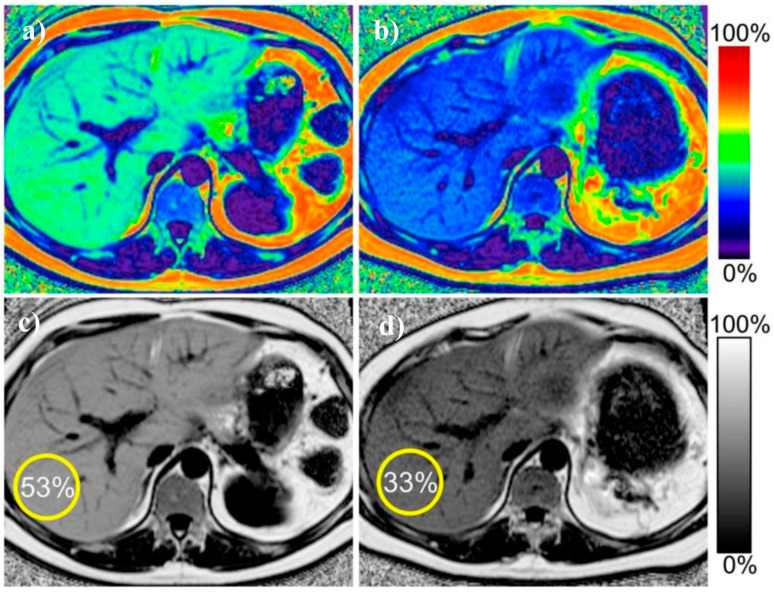
Example of proton density fat fraction liver maps in the same patient before and after treatment. Improvement in the liver steatosis and reduction in liver size is visible. (**a**) pre-treatment color-scale map (**b**) post-treatment color-scale map (**c**) pretreatment gray-scale map (**d**) post-treatment grey-scale map. Figure from Reeder SB, et al. Quantitative Assessment of Liver Fat with Magnetic Resonance Imaging and Spectroscopy. *J. Magn. Reson. Imaging* **2011**, *34*, 729–749 [43].

A recent meta-analysis by Gu et al., including studies with biopsy as the reference standard, has shown that MRI-PDFF provides excellent diagnostic accuracy, with a sensitivity of 93% for the detection of any grade of steatosis (defines as affecting at least 5% of hepatocytes) and a corresponding specificity of 94% [41]. Further, utilization of MRI-PDFF enables classification into different grades of hepatic steatosis with sensitivity and specificity of 74% and 87–90%, respectively, regarding the diagnosis of higher-grade steatosis. Another study performed by Middleton et al. has shown that MRI-PDFF performs well also in terms of monitoring patients during treatment, in particular in the assessment of discrete changes in liver steatosis in patients with decrease or increase in steatosis grade [42]. Importantly, the MRI-PDFF assessment was highly concordant with liver biopsy assessment regarding changes in liver histology, as 71% of patients with increasing steatosis were diagnosed as such with MRI-PDFF. Further, MRI-PDFF assessment showed an improvement in 91% of patients with decreasing steatosis. Only minor changes in MRI-PDFF assessment were noted in patients with stable histopathological findings. Figure 3 presents an example of two MRI-PDFF examinations in one patient, showing improvement in the degree of hepatic steatosis.

Although performing very well in terms of analyzing the amount of fat in the liver tissue, MRI-PDFF does not succeed in evaluating other variables that are clinically relevant. A study by Wildman-Tobriner et al. on patients taking part in clinical trials aimed at NAFLD/NASH has shown that the MRI-PDFF values overlap between patients with and without fibrosis, as well as between those with high and low NASH activity scores (NAS ≥ 4). Thus, MRI-PDFF does not seem to allow for discrimination between patients with mild and severe changes in histopathological examination [44]. Importantly, evaluation of steatosis using MRI-PDFF was compromised in case of concomitant fibrosis, with the correlation coefficient for the rate of hepatic steatosis and MRI-PDFF in patients with liver fibrosis of 0.60 as compared to the corresponding R of 0.86 in case of no fibrosis [45]. Therefore, MRI-PDFF should be cautiously interpreted in patients with either imaging or clinical suspicion of liver fibrosis.

**Figure 4:**
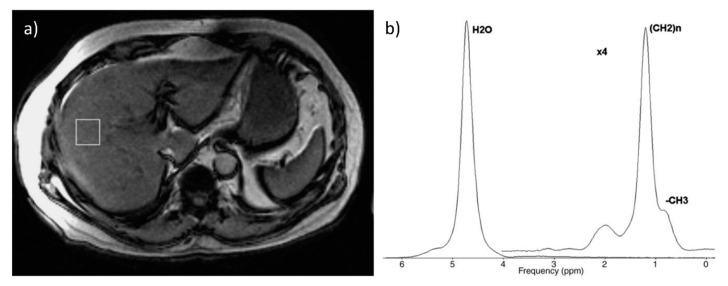
Example of spectroscopic examination: (**a**) voxel located in the right lobe (**b**) corresponding spectrum. Figure from Borra RJ, et al. Nonalcoholic fatty liver disease: rapid evaluation of liver fat content with in-phase and out-of-phase MR imaging. *Radiology* **2009**, *250*, 130–136 [57].

Magnetic resonance spectroscopy (MRS) allows us to calculate steatosis by directly measuring chemical composition of tissue in a chosen voxel, basing on the signal strength from each component (protons from water and fat) [46,47]. It is a well-established and accurate method of non-invasive liver fat quantification that has been validated and served as the reference standard in numerous studies [48,49,50,51,52,53,54]. A metanalysis performed by Bohte et al. (with histology used as a reference) has shown that the specificity of MRS in terms of detection of mild steatosis is 92% and increases up to 96% for the detection of more advanced fatty accumulation (>25%, >30% or >33%, depending on the study) [15]. These results are in line with a more recent study by Chiang et al., in which the MRS findings were compared to histological examinations in living liver donors with the reported sensitivity and specificity rates of the MR spectroscopy of 95% and 98%, respectively [55]. However, MRS requires sophisticated post-processing methods (spectral analysis), which substantially limits the accessibility to this method, as not every scanner is equipped with this modality. Figure 4 presents an example of liver spectroscopy, with a voxel representing the analyzed region and a corresponding spectrum. Moreover, in contrast to MRI-PDFF, which enables mapping of the whole organ, MRS analyses involve only a small portion of the liver parenchyma. The latter is therefore susceptible for sampling errors, similar to what liver biopsy is criticized for. The MRI-PDFF method is also less dependent on patient compliance. As the acquisition time for MRI-PDFF is shorter than that in the MRS, it is easier for the patient to remain still without breathing and it is also less time consuming in general [36,56].

**Figure 5:**
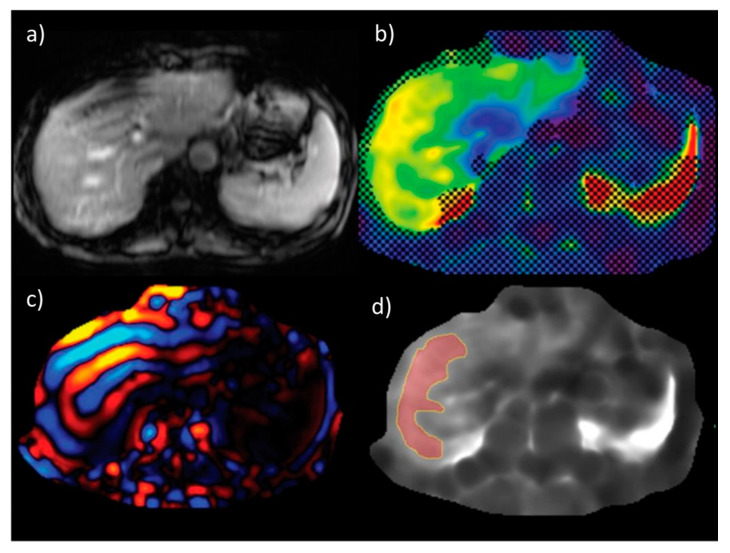
An example of liver stiffness measurement by magnetic resonance elastography (**a**) anatomic images (**b**) confidence map of an elastogram (**c**) wave image data (**d**) elastogram with free drawn region of interest. Figure from Chang, W., et al., Liver Fibrosis Staging with MR Elastography: Comparison of Diagnostic Performance between Patients with Chronic Hepatitis B and Those with Other Etiologic Causes. *Radiology* **2016**, *280*, 88–97 [62].

Magnetic resonance elastography (MRE) enables non-invasive assessment of hepatic fibrosis and is currently considered the most accurate non-invasive modality for its assessment, with a very good reproducibility and repeatability [58,59]. In a pooled analysis performed by Singh et al., the mean area under the receiver operating characteristics curve (with 95% confidence interval) values for diagnosing any (≥F1), significant (≥F2) or severe (≥F3) fibrosis and cirrhosis (F4) were 0.86 (0.82–0.90), 0.87 (0.82–0.93), 0.90 (0.84–0.94) and 0.91 (0.76–0.95), respectively [60]. These results were confirmed in another pooled analysis from 2020, by Liang Y. et al., in which the reported corresponding area under the receiver operating characteristics curve values were 0.89, 0.93, 0.93, and 0.95, respectively. The sensitivity rates in that study for of detection mild, significant, and severe liver fibrosis, and liver cirrhosis were 77%, 87%, 89%, and 94%, respectively, with the corresponding specificity rates of 90%, 86%, 84%, and 75%, respectively [61]. MRE also has its major disadvantages, including its high cost and insufficient availability, especially due to the fact that MR scanners are not regularly equipped with the elastography module. Nevertheless, MR elastography provides very good results and corresponds well to the fibrosis stage as assessed by the histopathological examination of liver biopsies. An example of magnetic resonance elastography is presented in Figure 5 [62].

The stiffness of healthy liver parenchyma in MRE is reported to be between 2.05 to 2.12 kPa [59], and the cut-off for normal liver stiffness is proposed to be set at 2.5 kPa [63]. However, the proposed cut-off values for discriminating between particular stages of fibrosis slightly differ among various studies and authors, with some authors also suggesting that the actual cut-off values may also be dependent upon the type of underlying liver disease. Chang et al., in their study on patients with chronic liver disease and healthy living liver donors, have shown that the MR elastography findings corresponded well with the stage of fibrosis with the areas under the receiver operating characteristics curve values ranging between 0.92 and 0.97. However, the actual liver stiffness value pointing towards the presence of cirrhosis remarkably differed between patients with hepatitis B virus infection (3.67 kPa) and those with other liver diseases (4.65 kPa) [62].

Liver stiffness can also be assessed by magnetic resonance with the use of diffusion weighted imaging (DWI); however, the results are inferior to MR elastography [64,65,66]. A meta-analysis by Wang et al. has shown that the sensitivity of MRE is 94% in detection of significant (≥F2) and 96% in detection of severe fibrosis (≥F2), remarkably higher than the corresponding values observed for DWI of 77% and 84%, respectively [66]. Some authors try to combine the DWI MR method with serum markers to increase the diagnostic accuracy, and the results show that this may be a reasonable alternative in cases where standard MRE is not available [67]. The authors of that study combined the DWI MR with the aspartate aminotransferase–to–platelet radio index (APRI) [22] and the Fibrosis-4 score, known as the FIB-4 [21]. This increased the diagnostic performance for discrimination between fibrosis grades 0–1 and 2–4: the area under the curve for DWI only was 0.72 and increased to 0.81 and 0.78 after addition of APRI and FIB-4, respectively. The performance to discriminate severe fibrosis (grades 0–2 versus 3–4) also increased −0.79 for DWI only, 0.83 for DWI + APRI, and 0.81 for DWI + FIB-4.

Standard abdominal MR examinations also allows the calculation of the amount of visceral and subcutaneous adipose tissue; however, due to the fact that the scanned area is usually limited to the liver and does not cover the whole abdominal cavity, it might be impossible to calculate all of the volumes (for example whole volume of visceral fat tissue).

## 4. Ultrasound Based Techniques

Liver steatosis can be detected on a regular B mode ultrasound; however, the diagnostic accuracy is low. Signs of liver steatosis on ultrasound typically comprise hyperechoic, bright liver (the echogenicity of the liver is usually compared to the echogenicity of the kidney), posterior attenuation or impaired visualization of intrahepatic vessels.

Studies have shown that the diagnosis of moderate or severe grades of liver steatosis by ultrasound is characterized by sensitivity and specificity rates of 80–91% and 87–98%, respectively. Nevertheless, these values drop to as low as 53% and 77%, respectively, when detecting steatosis of any grade [68,69,70,71,72].

Controlled attenuation parameter (CAP) is a relatively new method introduced by the Fibro-Scan. It measures liver attenuation to assess the degree of liver steatosis. The results are presented as dB/m (ranging from 100–400) and according to the manufacturers’ recommendation reflect the degree of steatosis [73,74]. This method has emerged relatively recently, with the first clinical studies published in 2010 [75], but has gained widespread acceptance and has been validated in numerous studies. Particularly good diagnostic accuracy of CAP was reported in a multicenter prospective study performed on the Chinese population (CAP measurements were compared with biopsy as a reference standard), with areas under the receiver operating characteristics curve of 0.92, 0.92 and 0.88 for detection of steatosis of at least 5%, 34%, and 67%, respectively [76]. These results are in line with another prospective study performed on Korean population, which reported the corresponding values for CAP of 0.885 for the detection of mild steatosis (sensitivity 73.1%, specificity 95.2%), 0.894 for detection of moderate steatosis (sensitivity 82.4%, specificity 86.1%) and 0.800 for detection of severe steatosis (sensitivity 77.8%, specificity 84.1%) [77]. The clinical utility of using CAP as a reference for the assessment of liver steatosis is largely limited by the low positive predictive value. In a study by Ferraioloi et al., the positive predictive values for the detection of mild or moderate steatosis using CAP cut-offs of 219 dB/M and 296 dB/M, respectively, were both below 60% despite relatively large areas under the receiver operating characteristics curve of 0.76 and 0.82, respectively [78]. A meta-analysis performed by Shi KQ et al. has consistently shown that CAP has good sensitivity and specificity, however the authors of the study concluded, that it should not be widely used as a standard method of steatosis assessment due to insufficient accuracy [79]. The positive and negative predictive vales of CAP ranged between 78–80% and 78–84%, depending on the stage of steatosis aimed for detection. Another meta-analysis by Karloas et al. has shown a good accuracy for the diagnosis of any or moderate steatosis with area under the receiver operating characteristics curve for >S0 and >S1 of 0.823 and 0.865, respectively [80]. A metanalysis by Pu et al. from 2019 also provided confirmation of acceptable sensitivity and specificity of CAP in detection of moderate and severe steatosis, yet pointed towards several factors, including age and body mass index, as potentially influencing its accuracy [81].

CAP has been shown to provide inferior diagnostic accuracy in comparison to MRI-PDFF in a study comparing these two methods with a biopsy reference: area under the receiver operating characteristics curve for MRI-PDFF in detection of any steatosis was 0.99 as compared to significantly lower value of 0.85 observed for CAP [82]. These results were also confirmed by another prospective study, which showed even lower value for CAP (0.77 versus 0.99 for MRI-PDFF) [83].

One of the biggest limitations of the use of CAP measurement was the M probe depth, which was not sufficient for obese patients. The manufacturer responded to that problem by introducing the XL probe, allowing for measurement in more overweight patients. The accuracy of measurements with the M and XL probe seem similar [84,85,86]. Although being an easy and relatively cheap method, CAP has also serious limitations when assessing patients with ascites and obesity, which substantially limits it use in the NAFLD/NASH patients, as vast majority of them is overweight.

**Figure 6:**
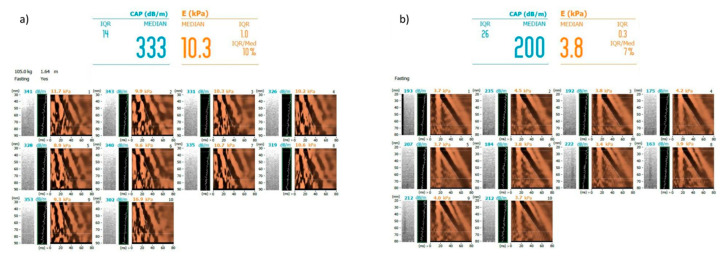
Examples of steatosis assessment by controlled attenuation parameter and liver stiffness assessment by transient elastography (Fibroscan) (**a**) in a patient with severe liver steatosis (grade 3) and severe fibrosis (F3) (**b**) in a patient with no liver steatosis (grade 0) and no liver fibrosis (F0). Images courtesy of Dr. Maciej Janik and Prof. Piotr Milkiewicz from the Liver and Internal Medicine Unit, Medical University of Warsaw.

Fibroscan device also allows the measurement of the liver stiffness by using the transient elastography technique (TE). There are some reports suggesting that although TE provides very good results, the accuracy in patients with NAFLD/NASH might be decreased. This is because the liver stiffness measurement can be affected by CAP values, particularly with respect to overestimation of the degree of liver fibrosis in the steatotic liver. Therefore, some authors have proposed different cut-off values for NAFLD patients when diagnosing fibrosis in TE [73,87]. In particular, high risk of false positive TE results with respect to detection of significant (F2–F4) and severe (F3–F4) liver fibrosis was noted for the liver stiffness ranges of 8.5–10.5 kPa and 10.1–12.5 kPa, respectively, in case of CAP of 300 to 339 dB/M, and 8.5–11.6 kPa and 10.1–13.6 kPa, respectively, in case of CAP exceeding 340 dB/M [73]. As both liver stiffness measurement by TE and steatosis measurement by CAP are available in the Fibroscan, the adjustment of the cut-off values can be done quickly and easily. However, the results of other studies are conflicting. In a recent study by Eddowes et al. performed on 450 patients with biopsy as reference, the accurate assessment of liver fibrosis and steatosis was reported with no negative influence of steatosis on the measurement of liver stiffness [88]. Although being a cheap, widely available, and easily performed technique, TE has inferior accuracy when comparing to magnetic resonance elastography. The area under the receiver operating characteristics curve for detection of any fibrosis (≥F1) using MRE was 0.82, which was significantly higher than that calculated for TE (0.67) [82]. Figure 6 presents an example of steatosis and fibrosis assessment (Fibroscan) with controlled attenuation parameters and transient elastography.

**Figure 7:**
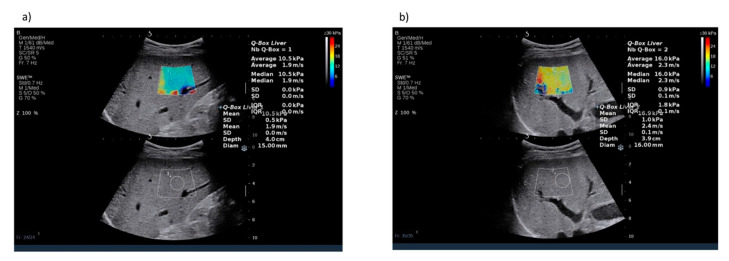
Examples of fibrosis assessment by shear wave elastography (**a**) in a patient with severe liver fibrosis (F3) (**b**) in a patient with cirrhosis (F4). Images courtesy of Dr. Maciej Janik and Prof. Piotr Milkiewicz from the Liver and Internal Medicine Unit, Medical University of Warsaw.

Another two methods of fibrosis assessment include the shear wave elastography (SWE) and acoustic radiation force impulse (ARFI), both of which can be integrated in regular ultrasound devices. SWE and ARFI have some notable disadvantages, such as the necessity to perform the measurement with patient holding breath. The results may also be influenced by the experience of the performing physician and in case of a recent food intake [89]. All the three methods provide results which are highly correlated with the fibrosis grade and have relatively good accuracy in detecting fibrosis. In a study comparing SWE, TE and ARFI performed by Casinotto et al., all three methods showed very similar diagnostic characteristics for the detection of corresponding grades of liver fibrosis [90]. Similar results were obtained by Lee et al., who also reported the similar ability of TE, SWE and ARFI in the diagnosis of liver fibrosis in a population of Asian NAFLD patients [91]. However, in another study, SWE was shown to provide superior results to TE in the accuracy of detecting any fibrosis, as well as discriminating between different fibrosis grades with areas under the receiver operating characteristics curve for SWE and TE for different fibrosis grades as follows: 0.86 and 0.80, respectively, for any fibrosis (≥F1); 0.88 and 0.78, respectively, for significant (≥F2) fibrosis; 0.93 and 0.83, respectively, for severe (≥F3) fibrosis; and 0.98 and 0.92, respectively, for cirrhosis (F4) [92]. Another study also showed superiority of SWE over TE and ARFI in diagnosing grade F2 or F3 of fibrosis, but without statistical difference regarding diagnosing mild fibrosis (F1) or cirrhosis (F4) [93]. Figure 7 presents an example of liver stiffness measurement by shear-wave elastography.

## 5. Dual-Energy X-ray Absorptiometry

Dual energy X-ray absorptiometry is a quick, relatively inexpensive and safe method of body composition assessment. Due to its clinical usefulness, it has gained wide acceptance and has been proposed in guidelines for the assessment of sarcopenia and obesity in the elderly population (European Working Group on Sarcopenia in Older People Consensus) [94,95]. Interestingly, some authors have proposed the implementation of special algorithms into the DXA examination to assess the liver fat amount. Bazzocchi et al. proposed placing a region of interest (ROI) in the location of the liver to calculate the amount of hepatic fat and have shown good correlation with liver steatosis assessed by biopsy (ρ = 0.610–0.619; *p* < 0.001), with an area under the curve ranging from 0.929 to 0.551 (depending upon sex and BMI category) [96].

## 6. Predictive Role of Imaging Methods in Patients with NAFLD

Patients with NAFLD are at high risk of developing systemic complications of the disease. This includes, in particular, the development of cardiovascular diseases and occurrence of cardiovascular events in case of underlying pathologies. In a 2014 study based on more than two thousand middle-aged adults without any known liver or heart disease, liver attenuation on computed tomography of less than 40 Hounsfield units, indicative of hepatic steatosis, was associated with approximately 30% more frequent occurrence of coronary artery calcifications and approximately 70% more frequent occurrence of abdominal aortic calcifications [97]. However, this significant association disappeared following adjustment for the amount of visceral fat, pointing towards the pivotal role of the latter as a major contributor to the development of atherosclerosis. Attenuation of the liver under 40 Hounsfield units on computed tomography scans was also found to be associated with subclinical cardiac remodeling and dysfunction in another population-based study, yet this was also largely attributable to increased amount of visceral adipose tissue [98]. These findings were recently supported by the results of the CARDIA study, in which the association between attenuation of the liver under 40 Hounsfield units and several structural and functional heart features lost significance following adjustment for obesity [99]. The same parameter predicted the presence of coronary microvascular dysfunction, which was an independent prognostic factor for the occurrence of a composite cardiovascular event end-point [100]. Notably, hepatic steatosis and fibrosis assessed on TE and CAP were similarly related to the presence of diastolic myocardial dysfunction in a study by Lee et al. [101]. Increased liver stiffness, as indicated by the results of SWE, increased the ability to predict the presence of coronary heart disease [102].

An analysis performed on 50 overweight adolescents revealed that intrahepatic fat content assessed on magnetic resonance spectroscopy was significantly associated with dyslipidemia independently of visceral fat content, as indicated by its positive correlation with plasma triglycerides, triglyceride to high-density lipoprotein ratio, or small dense low-density lipoprotein concentration, among others [103]. Further, the results of large cross-sectional Kangbuk Samsung Health Study comprising more than 100 thousand individuals pointed towards hepatic steatosis assessed on ultrasound as a significant predictor of the presence of coronary artery calcifications [104]. Increased prevalence of coronary artery calcifications in patients with ultrasound evidence for hepatic steatosis was independent of the presence of obesity.

Multiparametric evaluation of MR enabled prediction of the occurrence of liver-related clinical events in a study of 112 patients performed by Pavlides et al. [105]. More specifically, the use of liver inflammation and fibrosis score derived from analysis of T1 and T2 sequences led to the categorization of patients into subgroups based on the risk of developing clinical complications, with score values under 2 being characterized by a 100% negative predictive value. Additional MR analysis of hepatic iron and fat content further increased the predictive capacity of the model. In more than a thousand patients with severe liver fibrosis in the course of NAFLD, the baseline liver stiffness value was strongly associated with the occurrence of both decompensation of liver function and hepatocellular carcinoma, along with liver-related mortality [106]. Subgroup analysis from the same study performed by Petta et al. additionally provided evidence for the prognostic significance of the changes in liver stiffness measurement on TE by at least 20% with respect to those clinical outcomes and, furthermore, overall patient survival. Similarly, MRE-assessed liver stiffness exceeding 6.48 kPa was found to be predictive for the occurrence of liver function decompensation, with higher liver stiffness values also observed in patients with ascites, encephalopathy, and esophageal variceal bleeding [107].

## 7. Conclusions

**Figure 8:**
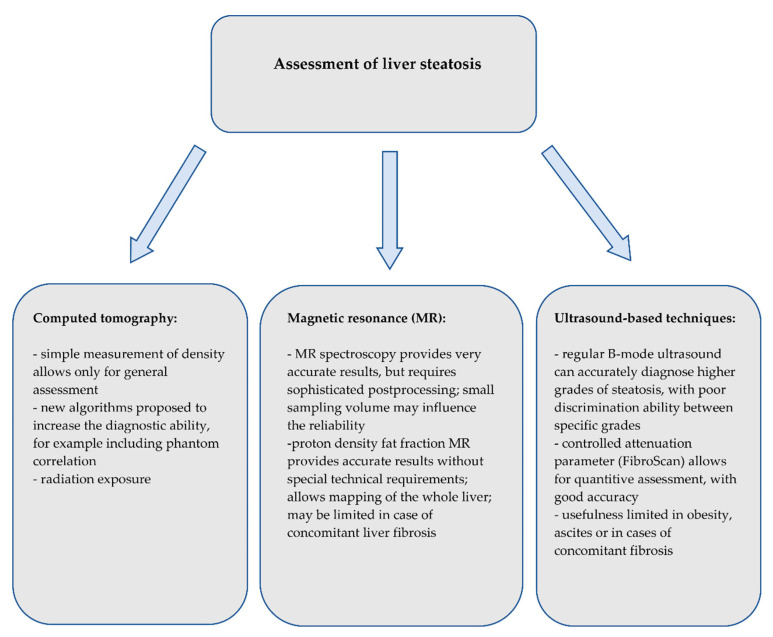
Summary of the imaging techniques for the assessment of liver steatosis.

**Figure 9:**
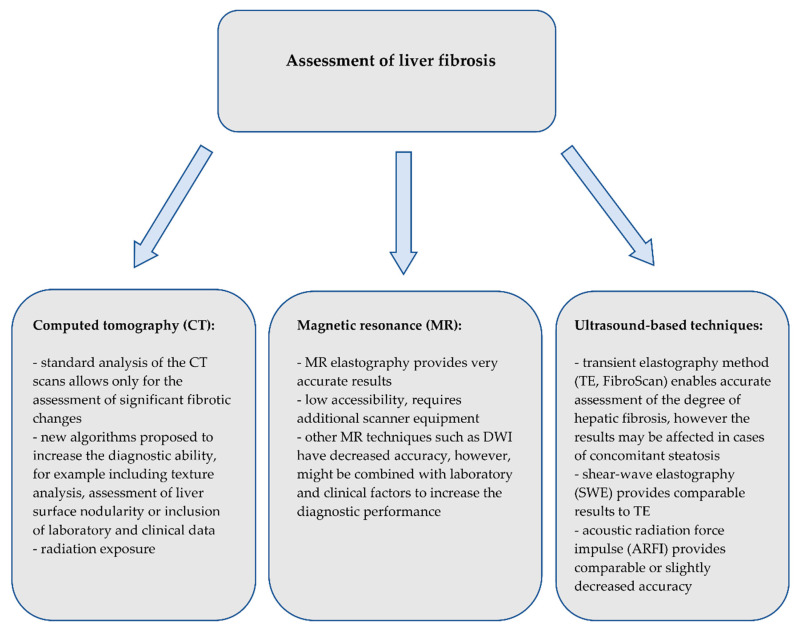
Summary of the imaging techniques for the assessment of liver fibrosis.

Although there are several noninvasive, accurate methods of the assessment of hepatic steatosis or fibrosis, liver biopsy is currently the only method that allows for the precise assessment of both, and moreover also the assessment of the inflammatory process. However, given that the prevalence of obesity, metabolic syndrome and NAFLD is and will be increasing in the upcoming years, alternative, widely accessible and noninvasive methods need to be introduced. Brief summary on the use of imaging techniques on detection of liver steatosis and fibrosis is shown on Figure 8 and Figure 9, respectively. Computed tomography only enables the diagnosis of higher grades of hepatic steatosis; however, new algorithms have been proposed to improve the diagnostic ability. Magnetic resonance spectroscopy provides highly reliable results, but its use is limited due to sophisticated postprocessing.

Proton density fat fraction MRI and the controlled attenuation parameter are the most promising techniques—MRI-PDFF with its ability to reliable quantify the fat percentage and the CAP, with low-cost machines, that can easily be used in outpatient clinics for initial screening purposes. Together with noninvasive methods of liver stiffness measurement, especially the magnetic resonance elastography or TE and SWE, those methods might be of crucial significance in distinguishing patients with moderate or severe changes for further assessment with liver biopsy. Computed tomography, magnetic resonance and transient elastography should be interpreted with respect to predicting patients’ clinical outcomes. Table 1 and Table 2 present a summary of diagnostic parameters of different methods for steatosis and fibrosis assessment.

## Figures and Tables

**Table 1:** Summary of the diagnostic parameters of different methods of steatosis assessment.

	Proposed Cut-Off	Sensitivity %	Specificity %
Computed tomography			
simple density measurement			
any steatosis (grade 1–3)	n/a	22–712 [14,15]	86–98 [14,15]
moderate steatosis (grade 2–3)	40 HU	60–82 [14,15]	88–98 [14,15]
phantom calibration [16]			
any steatosis (grade 1–3)	n/a	76% [16]	85% [16]
moderate steatosis (grade 2–3)	n/a	85% [16]	98% [16]
Magnetic resonance			
spectroscopy			
any steatosis (grade 1–3)	n/a	77–95 [15,53,55,56]	81–98 [15,53,55,56]
moderate steatosis (grade 2–3)	n/a	41–91 [15,53,56]	85–99 [15,53,56]
proton density fat fraction			
any steatosis (grade 1–3)	n/a	86–97 [35,36,41,42,43,44,45]	82–100 [35,36,41,42,43,44,45]
moderate steatosis (grade 2–3)	n/a	61–84 [35,36,41,42,43,44,45]	83–96 [35,36,41,42,43,44,45]
Ultrasound based techniques			
simple visual assessment			
any steatosis (grade 1–3)	subjective assessment	53–82 [15,68,69,70,71,72]	76–90 [15,68,69,70,71,72]
moderate steatosis (grade 2–3)		78–91 [15,68,69,70,71,72]	77–98 [15,68,69,70,71,72]
controlled attenuation parameter			
any steatosis	214–289 dB/m [76,77,78,79,80]	66–92 [76,77,78,79,80,81]	52–96 [76,77,78,79,80,81]
moderate steatosis	233–311 dB/m [76,77,78,79,80]	60–93 [76,77,78,79,80,81]	70–92 [76,77,78,79,80,81]

n/a—Not applicable; HU—Hounsfield Units.

**Table 2:** Summary of the diagnostic parameters of different methods of fibrosis assessment.

	Proposed Cut-Off	Sensitivity %	Specificity %
Computed tomography			
experimental algorithms			
any fibrosis (≥F1)	n/a	65–78 [18,19]	88–100 [18,19]
significant fibrosis (≥F2)	n/a	68–80 [18,19]	80–97 [18,19]
severe fibrosis (≥F3)	n/a	83–89 [18,19]	84–85 [18,19]
cirrhosis (F4)	n/a	90–98 [18,19]	80–85 [18,19]
Magnetic resonance			
elastography			
any fibrosis (≥F1)	1.77–5.02 kPa [60,61,62]	75–81 [60,61,62]	77–100 [60,61,62]
significant fibrosis (≥F2)	2.38–5.37 kPa [60,61,62,64]	79–97 [60,61,62,64]	81–100 [60,61,62,64]
severe fibrosis (≥F3)	2.43–5.97 kPa [60,61,62,64]	83–100 [60,61,62,64]	84–95 [60,61,62,64]
cirrhosis (F4)	2.74–6.7 kPa [60,61,62,64]	88–100 [60,61,62,64]	75–95 [60,61,62,64]
diffusion weighted imaging			
any fibrosis (≥F1)	n/a	75–86 [64,65,66]	71–94 [64,65,66]
significant fibrosis (≥F2)	n/a	67–92 [64,65,66]	61–91 [64,65,66]
severe fibrosis (≥F3)	n/a	48–90 [64,65,66]	65–100 [64,65,66]
cirrhosis (F4)	n/a	75–100 [64,65,66]	60–72 [64,65,66]
Ultrasound based techniques			
acoustic radiation force impulse			
any fibrosis (≥F1)	1.35 m/s [93]	61 [93]	96 [93]
significant fibrosis (≥F2)	0.95–1.38 m/s [90,91,93]	46–90 [90,91,93]	36–91 [90,91,93]
severe fibrosis (≥F3)	1.15–1.53 m/s [90,91,93]	59–90 [90,91,93]	63–90 [90,91,93]
cirrhosis (F4)	1.3–2.04 m/s [90,91,93]	44–90 [90,91,93]	67–90 [90,91,93]
transient elastography			
any fibrosis (≥F1)	6.7–8 kPa [92,93]	65–83 [92,93]	83–91 [92,93]
significant fibrosis (≥F2)	6.2–9.8 kPa [90,91,92,93]	60–90 [90,91,92,93]	45–92 [90,91,92,93]
severe fibrosis (≥F3)	8–12.5 kPa [90,91,92,93]	57–90 [90,91,92,93]	61–92 [90,91,92,93]
cirrhosis (F4)	9.5–16.1 kPa [90,91,92,93]	65–92 [90,91,92,93]	62–92 [90,91,92,93]
shear wave elastography			
any fibrosis (≥F1)	6.5–7.8 kPa [92,93]	68–84 [92,93]	91–100 [92,93]
significant fibrosis (≥F2)	6.3–8.7 kPa [90,91,92,93]	71–90 [90,91,92,93]	50–92 [90,91,92,93]
severe fibrosis (≥F3)	8.3–10.7 kPa [90,91,92,93]	71–91 [90,91,92,93]	71–90 [90,91,92,93]
cirrhosis (F4)	10.1–15.1 kPa [90,91,92,93]	58–97 [90,91,92,93]	72–93 [90,91,92,93]

n/a—Not applicable.

## Abbreviations

NAFLD	non-alcoholic fatty liver disease
NASH	non-alcoholic steatohepatitis
MRS	magnetic resonance spectroscopy
MRI-PDFF	magnetic resonance imaging proton density fat fraction
CAP	controlled attenuation parameter
MRE	magnetic resonance imaging
TE	transient elastography
SWE	shear wave elastography
ARFI	acoustic radiation force impulse
CT	computed tomography
BMI	body mass index
HCC	hepatocellular carcinoma
NAS	NASH activity score
DWI	diffusion weighted imaging
APRI	aminotransferase-to-platelet ratio index
FIB-4	Fibrosis-4 score
